# A Deep Learning Approach to Population Structure Inference in Inbred Lines of Maize

**DOI:** 10.3389/fgene.2020.543459

**Published:** 2020-11-24

**Authors:** Xaviera Alejandra López-Cortés, Felipe Matamala, Carlos Maldonado, Freddy Mora-Poblete, Carlos Alberto Scapim

**Affiliations:** ^1^Department of Computer Sciences and Industries, Catholic University of the Maule, Talca, Chile; ^2^Instituto de Ciencias Agroalimentarias, Animales y Ambientales, Universidad de O’Higgins, San Fernando, Chile; ^3^Institute of Biological Sciences, University of Talca, Talca, Chile; ^4^Departamento de Agronomia, Universidade Estadual de Maringá, Maringá, Brazil

**Keywords:** deep learning, genome-wide studies, machine learning, single-nucleotide polymorphisms, dimensionality reduction

## Abstract

Analysis of population genetic variation and structure is a common practice for genome-wide studies, including association mapping, ecology, and evolution studies in several crop species. In this study, machine learning (ML) clustering methods, K-means (KM), and hierarchical clustering (HC), in combination with non-linear and linear dimensionality reduction techniques, deep autoencoder (DeepAE) and principal component analysis (PCA), were used to infer population structure and individual assignment of maize inbred lines, i.e., dent field corn (*n* = 97) and popcorn (*n* = 86). The results revealed that the HC method in combination with DeepAE-based data preprocessing (DeepAE-HC) was the most effective method to assign individuals to clusters (with 96% of correct individual assignments), whereas DeepAE-KM, PCA-HC, and PCA-KM were assigned correctly 92, 89, and 81% of the lines, respectively. These findings were consistent with both Silhouette Coefficient (SC) and Davies–Bouldin validation indexes. Notably, DeepAE-HC also had better accuracy than the Bayesian clustering method implemented in InStruct. The results of this study showed that deep learning (DL)-based dimensional reduction combined with ML clustering methods is a useful tool to determine genetically differentiated groups and to assign individuals into subpopulations in genome-wide studies without having to consider previous genetic assumptions.

## Introduction

Analysis of population structure and genetic variation is a common practice in genome-wide studies and is an important guideline to understand and infer the evolutionary processes and the demographic history in ecological and evolutionary studies (Stift et al., 2019). Knowledge of the population genetic structure is very helpful in many applications, which plays an important role for breeding purposes and selection strategies. In this sense, high-throughput DNA sequencing technologies have allowed the generation of large sets of genomic data in diverse populations routinely (Ho et al., 2019), which has been used to study patterns of genetic variation across the genome and to characterize the evolutionary forces in different plant species (Padhukasahasram, 2014; Ho et al., 2019). For instance, markers based on single-nucleotide polymorphisms (SNPs) have provided a rapid way of delineating genetic structure and of understanding the basis of the taxonomic discrimination, providing novel information such as founder effects, bottlenecks, evolutionary relationships, and migration history of natural populations (Padhukasahasram, 2014; Shultz et al., 2016).

Population structure analysis is a major area of interest within the field of genetics and bioinformatics (Alhusain and Hafez, 2018). In this sense, several bioinformatics methods have been developed to examine the population structure in genetically diverse plant germplasm based on high-throughput genomic data. Among the methods currently available, the Bayesian clustering algorithm developed by Pritchard et al. (2000) (i.e., STRUCTURE) is one of the most widely used population analysis tools, which allows researchers to infer population structure patterns in sample sets (Porras-Hurtado et al., 2013). The underlying genetic model of this algorithm assumes that populations are in Hardy–Weinberg equilibrium (Pritchard et al., 2000), which is not met, for instance, in populations with high levels of inbreeding. In this sense, Gao et al. (2007) proposed an extension to the STRUCTURE algorithm denominated InStruct, which eliminates the assumption of Hardy–Weinberg equilibrium within populations and takes inbreeding or selfing into account. This method applies a Bayesian inference to simultaneously assign individuals into subpopulations but can be very time-consuming. Another successful approach to infer population structure has been implemented in the ADMIXTURE software (Alexander et al., 2009; Alexander and Lange, 2011), a maximum-likelihood-based method that updates the log-likelihood as it converges on a solution for the ancestry proportions and allele frequencies that maximize the likelihood function (Alexander and Lange, 2011). Other authors have emphasized the use of non-parametric methods such as K-means (KM) and hierarchical clustering (HC) (Bouaziz et al., 2012; Meirmans, 2012; Alhusain and Hafez, 2018). KM and HC approaches correspond to machine learning (ML) methods that do not require the assumptions of the Hardy–Weinberg principle and use external dimension reduction techniques, such as principal component analysis (PCA) (Kobak and Berens, 2019), commonly used in several data-intensive biological fields. KM is an iterative descent algorithm that minimizes the within-cluster sum of squares (Meirmans, 2012). On the other hand, the HC method allows the formation of genetic groups to be mutually exclusive, in which each cluster is distinct from each other, and the members of each cluster are similar with respect to the input information (Ward, 1963). Stift et al. (2019) found that ADMIXTURE and KM were computationally faster than STRUCTURE; however, ADMIXTURE had less power to detect structure compared to STRUCTURE and KM clustering.

The analytical Bayesian inference-based methods (STRUCTURE and InStruct) and the most traditional ML algorithms require that the data provided need to be of numerical type (Pritchard et al., 2000; Yokota and Wu, 2018). Label encoder (LE) is a useful method to help normalize labels so that they can transform non-numerical values into numerical values (Joshi et al., 2016). In genomic data, for instance, Agajanian et al. (2019) used LE to assign to each nucleotide a unique numeric data value. Other ML methods [e.g., deep autoencoder (DeepAE), a likelihood-free inference framework] consider a framework in which the information of the input variables is compressed and subsequently reconstruct the input data, minimizing the loss function. In this sense, the deep learning (DL) approach is a class of neural networks and has been an active area of ML research, emerging as a powerful tool in genetics and genomics studies, e.g., schizophrenia classification through datasets of SNP and functional magnetic resonance imaging (Li et al., 2020), gene expression prediction from SNP genotypes (Xie et al., 2017), MADS-box gene classification system for angiosperms (Chen et al., 2019), and RNA secondary structure prediction (Zhang et al., 2019) and to predict quantitative phenotypes from SNPs (Liu et al., 2019). Unlike the traditional artificial neural network, DL algorithms consider many hidden layers during the network training (Xie et al., 2016). The advantages of the DL approach have been well described by Qu et al. (2019) and can be summarized as the capacity of (1) learning from data without prediction features, (2) learning from increasingly large and high-dimensional datasets, and (3) capturing non-linear dependencies in genetic sequences. Therefore, in this study, a genome-wide data assessment of maize inbred lines was performed using the DL (DeepAE) approach, combined with ML methods (HC and KM) and a Bayesian clustering approach (InStruct), to infer population genetic structure and assign individuals into each subpopulation. A better understanding of the use of these novel methods could provide recommendations for genetic diversity and differentiation studies.

## Materials and Methods

**FIGURE 1:**
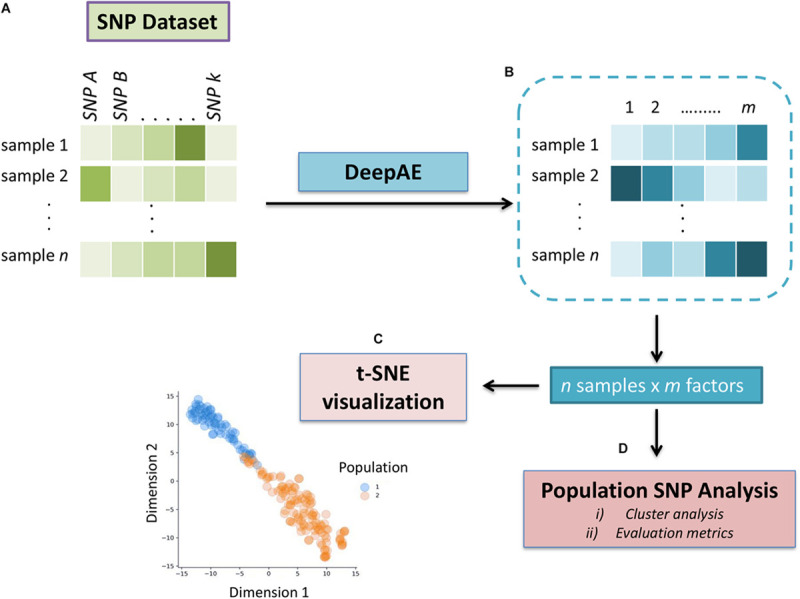
Overview of the deep autoencoder (DeepAE) system and population clustering analysis from a large single-nucleotide polymorphism (SNP) dataset. **(A)** Schematic representation of the entity system. Input data are composed of a matrix of n samples (rows) and k SNPs (columns). Through autoencoder, categorical variables, i.e., the SNPs and samples, were represented as an m-dimensional vector. **(B)** Samples are projected into m dimensional sample entity vector space. The DeepAE learned the feature of the sample solely from input matrix, such that similar samples are clustered in close proximity. **(C)** The t-distributed stochastic neighbor embedding (t-SNE) representation of sample entity matrix from the SNP dataset transformed by DeepAE. The 183 inbred lines are labeled with a different color according to subpopulations of maize (dent corn and popcorn). **(D)** Population clustering analysis through unsupervised clustering techniques.

The step-by-step description of the proposed methodology for the clustering of populations, through the use of DL and ML, is illustrated in Figure 1. The respective codes are available in Supplementary Data Sheet S1. The first step is to preprocess SNP dataset and apply DeepAE with two layers in both the encoder and decoder, without considering the input and output. The second step is based on applying ML clustering algorithms in an unsupervised way based on the data obtained from the DeepAE in order to group and identify subpopulations.

### Genotyping and Data Processing

These inbred lines correspond to a panel of 183 maize genotypes from the Department of Agronomy of the State University of Maringá, which consist of 97 dent field corn and 86 popcorn genotypes (for more details, see Supplementary Data Sheet S2). Seedlings were grown in a growth chamber at 27/20°C day/night temperatures and a 12-h photoperiod. The youngest leaves of five plants were sampled from each genotype ∼30 days after germination. The DNA samples were sent to the Genomic Diversity Institute of Cornell University for SNP discovery *via* genotyping-by-sequencing (GBS), which is described in Elshire et al. (2011). The TASSEL 5.2 software (Bradbury et al., 2007) was used to align the raw data of GBS with the *Zea mays* version AGPV3 reference genome (B73 RefGen v3), resulting in a total of 1,014,070 SNPs. Subsequently, these SNPs were filtered through TASSEL considering a minor allele frequency > 0.15 and the absence of missing data, yielding a final subset of 4,812 SNPs (distributed on all chromosomes).

### Dimensionality Reduction Methods

#### Unsupervised Learning Using Deep Autoencoder

DeepAE was applied to find a mathematical representation of SNPs and to reduce the dimensionality of the dataset. This architecture contains multiple encoding and decoding stages made up of a sequence of encoding layers followed by a stack of decoding layers. First, the SNPs were encoded to a numerical representation through one hot encoding process, as follows: A: [1,0,0,0], T: [0,1,0,0], G: [0,0,1,0], C: [0,0,0,1]. The depth of the network was varied from one to four hidden layers in order to minimize the loss function (cross-entropy function; Supplementary Data Sheet S3), in which the best results were obtained considering two hidden layers. Therefore, DeepAE was performed considering the following parameters: an entrance of 4,812 features corresponding to SNP markers (represented by one hot encode), two hidden layers with 2,000 and 700 neurons, respectively, in both the encoder and decoder, a bottleneck hidden layer of 40 neurons, and a learning rate of 0.001. Details about DeepAE are shown in Supplementary Data Sheet S3. The Adam optimizer was used to minimize the loss function (cross-entropy function). The rectified linear unit (ReLU) was used as the activation function. DeepAE was implemented in python 3.7 language using the libraries Keras 2.2.4 and TensorFlow 1.14.0.

#### Principal Component Analysis

The PCA describes the variation of a dataset in terms of a set of uncorrelated variables, where each of these is a linear combination of the original variables. These new variables are sorted in descending order of importance, where the first variable (or first principal component) accounts for a majority of the variation in the original data, and the following variables account for a large amount of the remaining variation of the data that is not correlated with the previous variables. The PCA was performed in TASSEL 5.2 (Bradbury et al., 2007).

### Visualization of Reduced Data by Deep Autoencoder

The t-distributed stochastic neighbor embedding (t-SNE) is a technique that allows visualization of high-dimension data giving each data point a location in a low dimension (Maaten and Hinton, 2008). This method maps the different high-dimension instances into new low-dimension instances keeping up the similarities found in the original data. Encoded SNPs with DeepAE were visualized by two-dimensional t-SNE implemented with perplexity = 30, iterations equal to 1,000, and a learning rate of 200.

### Clustering Analysis

Three types of unsupervised clustering algorithms were applied: KM (Macqueen, 1967), HC (Abbas, 2008), and InStruct (Gao et al., 2007). Details about these three clustering algorithms are shown in Supplementary Data Sheet S3. The entrance for these methods corresponds, on one hand, to the SNP genomic data represented with LE and, on the other hand, to the encoded SNP data with dimensionality reduction techniques: DeepAE and PCA. Specifically, LE is a numerical representation to transform non-numerical labels to numerical labels (Joshi et al., 2016); in this case, the SNP markers were processed as follows: A:[0], T:[1], G:[2], C:[3]. The genomic data represented by LE and the dimension reduction techniques were used as inputs in the ML clustering methods, while the Bayesian method only used the dataset codified with LE (Supplementary Figure S4). The optimal number of clusters was determined by two validation metrics: Silhouette coefficient (SC; Rousseeuw, 1987) and Davies–Bouldin index (DBI; Davies and Bouldin, 1979) for the ML-based clustering algorithms (details about evaluation metrics are shown in Supplementary Data Sheet S3). On the other hand, the optimal number of clusters (K) in Bayesian-based clustering algorithm (InStruct) was determined with the highest ΔK method, as proposed by Evanno et al. (2005), and the lowest value of deviance information criterion (DIC) (Gao et al., 2007).

## Results

### Population Clustering Analysis With K-Means, Hierarchical Clustering, and InStruct

The results of clustering analysis with KM and HC varied depending on the data preprocessing algorithms being studied (i.e., LE, DeepAE, and PCA). The results of KM and HC methods showed that LE was less accurate than DeepAE and PCA according to SC and DBI measures (Table 1). In fact, these validation indexes (SC and DBI) were ∼10 and ∼8 times higher for DeepAE and PCA, respectively, than LE, when K = 2. The SC values showed that the reliability of clusters generated by the three data preprocessing algorithms decreases as the amount of K clusters increases (Table 1), achieving the best accuracy measures for K = 2. Consistently, in the three preprocessing algorithms, the DBI was higher when the number of clusters increased. Moreover, KM and HC in combination with DeepAE and PCA showed the best results in terms of accuracy when K = 2. The high values of SC obtained for PCA and DeepAE in combination with both clustering methods (close to 1; Table 1) indicate that an inbred line is well matched to its own genetic cluster and poorly matched to the neighboring group or subpopulation. On the other hand, the SC value for LE in combination with HC (LE-HC) was close to zero and achieved the same value for K = 2, 4, and 5, while DBI for LE-HC revealed that the optimal number of clusters was K = 4. According to these results, through the classical representation (LE), it was not possible to achieve a consistent clustering performance with neither ML clustering method (KM and HC), thus this representation was discarded from the posterior cross-tab analysis. In the case of DeepAE or PCA, it was possible to achieve the optimal number of clusters.

**TABLE 1:** Validation indexes for the optimal number of clusters (K) according to Silhouette coefficient (SC) and Davies–Bouldin index (DBI).

**K**	**K-means**						**Hierarchical clustering**					
	**LE**		**PCA**		**DeepAE**		**LE**		**PCA**		**DeepAE**	
	**SC**	**DBI**	**SC**	**DBI**	**SC**	**DBI**	**SC**	**DBI**	**SC**	**DBI**	**SC**	**DBI**
2	0.08	2.93	0.67	0.39	0.78	0.30	0.08	2.94	0.67	0.34	0.78	0.30
3	0.05	3.59	0.61	0.55	0.74	0.39	0.07	2.69	0.65	0.38	0.73	0.38
4	0.05	3.58	0.56	0.55	0.57	0.59	0.08	2.52	0.59	0.49	0.59	0.55
5	0.04	3.53	0.56	0.66	0.51	0.62	0.08	2.72	0.52	0.5	0.56	0.55
6	0.05	3.79	0.48	0.69	0.51	0.61	0.05	2.95	0.42	0.54	0.48	0.58
7	0.05	3.71	0.47	0.69	0.49	0.62	0.05	2.92	0.46	0.69	0.47	0.63
8	0.06	3.37	0.37	0.81	0.47	0.67	0.06	3.35	0.38	0.64	0.46	0.63
9	0.06	3.49	0.39	0.82	0.44	0.68	0.06	3.15	0.37	0.63	0.44	0.71

*The clusters were formed by machine learning (ML) clustering methods, K-means, and hierarchical clustering, in combination with the following data preprocessing algorithms: label encoder (LE), principal component analysis (PCA), and deep autoencoder (DeepAE).*

The Bayesian clustering analysis with InStruct indicated that the 183 inbred lines were grouped into two clusters (K = 2) according to the lowest DIC and the highest second-order change rate of the probability function with respect to K (ΔK). This result was expected, since the inbred lines come from two well-defined maize subpopulations (i.e., dent corn and popcorn), which was confirmed by DIC and ΔK values obtained from InStruct and both SC and DBI validation measures in the clusters formed by both ML clustering methods in combination with DeepAE and PCA (Table 1). In this study, the majority of the dent corn lines were grouped in cluster 1, whereas the majority of popcorn lines were assigned to cluster 2.

A simple cross-tab analysis was performed to evaluate the ability of clustering and preprocessing methods to assign individuals to their putative subpopulation (i.e., dent corn and popcorn). The results of this analysis are shown in Table 2 for KM and HC ML clustering algorithms in combination with DeepAE and PCA dimension reduction algorithms and the Bayesian clustering method implemented in InStruct. DeepAE combined with both HC (DeepAE-HC) and KM (DeepAE-KM) methods grouped the smallest amount of popcorn lines within cluster 1 (which should be composed of only dent corn lines). The Bayesian approach implemented in InStruct grouped 17 popcorn lines within cluster 1, while PCA combined with HC (PCA-HC) and KM (PCA-KM) grouped the greatest amount of popcorn lines together with dent corn lines (cluster 1) (Table 2). Interestingly, the SC validation index was higher in DeepAE than PCA (for both clustering methods), which implies that average within-cluster distances were low (high compactness in clusters), whereas between-cluster distances were high (high separation between clusters). It should be noted that the Bayesian clustering method (InStruct) and ML clustering algorithms (HC and KM) grouped coincidently eight popcorn lines into cluster 1, i.e., the cluster containing dent corn lines. Overall, DeepAE-HC was the most effective method to assign individuals to the clusters (96% of correct individual assignments), whereas DeepAE-KM, PCA-HC, PCA-KM, and InStruct assigned correctly 92, 89, 81, and 91%, respectively, of the lines into their respective clusters (Table 2).

**TABLE 2:** Cross-tab analysis among subpopulations of maize (popcorn and dent corn) and clusters predicted by machine learning (ML) clustering methods in combination with dimensionality reduction techniques.

**Methods**	**Predicted**	**Cluster 1**	**Cluster 2**	%CA*
DeepAE-KM	Cluster 1	97	15	92%
	Cluster 2	0	71	
DeepAE-HC	Cluster 1	97	8	96%
	Cluster 2	0	78	
PCA-KM	Cluster 1	97	34	81%
	Cluster 2	0	52	
PCA-HC	Cluster 1	97	20	89%
	Cluster 2	0	66	
InStruct	Cluster 1	97	17	91%
	Cluster 2	0	69	

Clusters 1 and 2 represent the dent field corn (*n* = 97) and popcorn (*n* = 86) subpopulations of maize, respectively. *Percentage of inbred lines of maize correctly assigned to the clusters.

### Visualization of the Genetic Structure With t-Distributed Stochastic Neighbor Embedding and Principal Component Analysis

**FIGURE 2:**
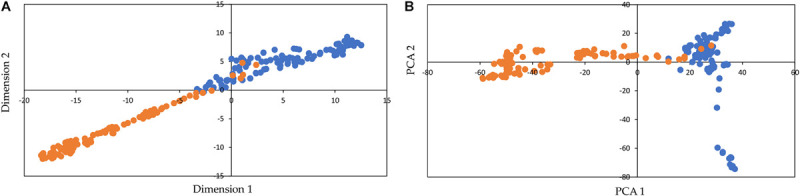
Visualization of the genetic structure of inbred lines of maize with t-distributed stochastic neighbor embedding (t-SNE) for the DeepAE **(A)** and representation of principal component analysis (PCA) **(B)** for single-nucleotide polymorphism (SNP) data. The colored dots orange and blue represent different inbred lines from the two subpopulations of maize (dent corn and popcorn, respectively).

Figure 2 shows the visualization with t-SNE (for DeepAE) and the PCA representation for SNP data. t-SNE and PCA clearly separated the inbred lines into two clusters, which correspond to the subpopulations of popcorn (blue) and dent corn (orange) (Figure 2). The t-SNE method allowed to define the clusters with any of its dimensions (1 and 2), while the PCA only separated the clusters with dimension 1. Moreover, t-SNE clustered the individuals of each putative subpopulation closer together than the PCA method. In this sense, t-SNE preserved the local structure (more than the larger-scale structure) of data by matching pairwise similarity distributions in the higher-dimensional space (original data) in a lower-dimensional projected space (Chan et al., 2018), and thus, as opposed to PCA, t-SNE grouped the samples in a low-dimensional space, while keeping the distributions of original data space.

## Discussion

Analysis of population genetic structure is a major area of interest within the field of genetics and bioinformatics, which is a common practice in genome-wide studies, including association mapping, ecology, and evolution studies in crop species such as maize (Li et al., 2019; Mafra et al., 2019; Maldonado et al., 2019; Wang et al., 2019). The present study proposed an ML-based analysis of population structure and individual assignment usually performed in several data-intensive biological fields. According to the results, HC in combination with the two data preprocessing algorithms (DeepAE and PCA) presented higher accuracy in assigning maize lines to their respective clusters as compared to KM. These findings agree with a previous study by Kaur and Kaur (2013), who reported that the hierarchical algorithm provides better results and higher quality than KM. Additionally, the results of this study were consistent with the findings of previous research, indicating that dent corn and popcorn lines from Brazilian germplasms are grouped into two genetically differentiated clusters (Coan et al., 2018; Camacho et al., 2019; Maldonado et al., 2019; Senhorinho et al., 2019). In this regard, the results of this study showed that the DeepAE-based data preprocessing had better accuracy values than those achieved by PCA. In this sense, PCA-HC and PCA-KM had a high number of lines incorrectly assigned to cluster 1 (20 and 34, respectively). On the other hand, InStruct showed an accuracy value lower than DeepAE-HC and DeepAE-KM when assigning maize lines. In this sense, Stift et al. (2019) found that InStruct revealed more inconsistency than KM in the clustering results, which was derived from a lack of convergence across replicate runs of the algorithm.

The conventional clustering methods, e.g., self-organizing map algorithm (Kohonen and Somervuo, 1998), Gaussian mixture models (Reynolds, 2015), KM (Arthur and Vassilvitskii, 2007), and HC (Bouaziz et al., 2012), usually have poor clustering performance on high-dimensional data due to high computational complexity (Min et al., 2018). For this reason, dimensionality reduction methods have been widely studied to represent the raw data into a low-dimensional space to ensure that the data are easier to separate when using clustering methods. The most popular methods for dimensionality reduction include linear transformation with PCA and non-linear transformation with autoencoder (Vincent et al., 2008; Chazan et al., 2019; Kobak and Berens, 2019). However, the non-linear nature of an autoencoder has been shown to reconstruct complex data more accurately than PCA (Xie et al., 2016). Sakaue et al. (2020) pointed out that the main linear technique for dimensionality reduction, PCA, was not sufficient to fully capture the fine and subtle genomic structure within a Japanese population (*n* = 169,719), while non-linear dimensionality reduction methods (t-SNE and uniform manifold approximation and projection) could detect a fine and discrete population structure with a high resolution. Tan et al. (2014) showed that the use of denoising autoencoders was efficient to identify and extract complex patterns from a large collection of breast cancer gene expression data, which allowed for successfully constructed features that contain both clinical and molecular information. In this sense, Yue and Wang (2018) pointed out denoising autoencoders are effective in extracting biological insights, since the reconstruction loss of autoencoder ensures that the network learns a feasible representation and avoids obtaining trivial solutions. On the other hand, Almotiri et al. (2017) showed that the non-linear autoencoder achieved better accuracy than the linear PCA method in the classification of handwritten numerals. In accordance with the present study, Manning-Dahan (2018) observed that autoencoder had an accuracy 68% higher than PCA, with much less false positives found in the classification of images. This author also pointed out that PCA creates linear maps and, thus, is limited to learn linear relationships between variables, whereas autoencoders can be used for encoding and decoding large datasets with the flexibility of learning both non-linear and linear mappings. Xie et al. (2016) also pointed out that KM has a better performance when it is employed on a set of data preprocessed by autoencoder than when they have not been preprocessed. Therefore, a key aspect of the methodology proposed in this study is the correct mathematical representation of the SNP dataset, which is not achieved with a classical technique such as LE or PCA but is achieved through the implementation of more complex techniques, such as DeepAE.

Artificial neural network models have been used before in order to genetically evaluate crop germplasm collections, such as maize (Ferreira et al., 2018; Kulka et al., 2018) and grapevine (Costa et al., 2020), in which the clustering analysis was based upon competitive learning-based neural networks. This alternative method was able to analyze population structure based on not only bi-allelic but also multi-allelic data (Peña-Malavera et al., 2014; Ferreira et al., 2018) and has been demonstrated to be computationally faster than MCMC methods (Nikolic et al., 2009) and does not consider the assumption of Hardy–Weinberg equilibrium in the population being studied (Ferreira et al., 2018). In this sense, the ML algorithms required less time for its run as compared to InStruct. ML clustering algorithms in combination with DeepAE or PCA dimension reduction algorithms require approximately 3 s for execution, while with InStruct, the time required was about 3 weeks. InStruct is based on the Markov chain method for parameter estimation, which is computationally time-consuming with respect to other unsupervised clustering methods (Gao et al., 2007; Stift et al., 2019). It should be noted that the artificial neural networks have the advantage of being a non-parametric method, which does not require detailed information about the physical processes being modeled and is able to analyze data containing missing data (Azevedo et al., 2015; Costa et al., 2020). Interestingly, our results confirm that DeepAE neural networks provide precise results in the identification of genetically differentiated groups and the assignment of lines into subpopulations (Table 2).

On the other hand, the t-SNE algorithm, in combination with DeepAE data, was able to visually identify both subpopulations of maize (Figure 2). This is of great relevance since it is the starting point in the unsupervised clustering algorithms and identifying clusters. Kobak and Berens (2019) pointed out that when applied to high-dimensional but well-clustered data, t-SNE tends to produce a visualization with distinctly isolated clusters, which often are in agreement with the clusters found by a dedicated clustering algorithm. Also, these authors mentioned that the combination of t-SNE with a variational autoencoder better preserves the global structure of the data and produces more interpretable visualizations than standard t-SNE. In this sense, this study found that t-SNE was better than PCA in preserving the local structure by grouping genotypes of each putative subpopulation closer together. Moreover, t-SNE could separate the subpopulations with any dimension (1 and 2), while a PCA separated the subpopulations only with the first dimension.

Finally, the use of the novel dimensionality reduction method, DL, combined with ML clustering methods allowed to assign popcorn and dent corn lines into their respective maize subpopulations. This analytical methodology can be applied to uncover the genetic structure in diverse populations worldwide, without having to consider previous genetic assumptions such as Hardy–Weinberg and linkage disequilibrium.

## Data Availability Statement

Publicly available datasets were analyzed in this study. These data can be found here: https://doi.org/10.6084/m9.figshare.12934913.

## Author Contributions

FM-P, CM, CS, and XL-C conducted and designed this study. CM, XL-C, and FM implemented the database and web application. FM-P, CM, and CS performed the data curation. FM-P, CM, and XL-C wrote the manuscript. All authors reviewed and approved the manuscript for publication.

## Conflict of Interest

The authors declare that the research was conducted in the absence of any commercial or financial relationships that could be construed as a potential conflict of interest.
