# Supplementary material for: A Deep Learning Approach to Population Structure Inference in Inbred Lines of Maize
Source: Front Genet. 2020 Nov 24;11:543459. doi: 10.3389/fgene.2020.543459 (PMC7732446; doi:10.3389/fgene.2020.543459)
Supplement: Supplementary file 1 [file Data_Sheet_1.ZIP › Supplementary_Data_Sheet_S1.docx]

from sklearn.metrics import silhouette_score, davies_bouldin_score

from sklearn.cluster import KMeans, AgglomerativeClustering

from sklearn.model_selection import train_test_split

from keras.layers import Input, Dense

from keras.models import Model

import numpy as np

import pandas as pd

################################ READ DATA #################################

dataset = pd.read_csv("dataset_snp.csv", sep=";")

X = np.array(dataset.drop(['Population '], axis=1))

############################### TRAIN AND TEST SPLIT #########################

X_train, X_test = train_test_split(X, test_size=0.1)

############################# DEEP AUTOENCODER #############################

vector_length = X.shape[1]

placeholder_input = Input(shape=(vector_length,))

encoded = Dense(2000, activation='relu')(placeholder_input)

encoded = Dense(700, activation='relu')(encoded)

encoded = Dense(40, activation='relu')(encoded)

decoded = Dense(700, activation='relu')(encoded)

decoded = Dense(2000, activation='relu')(decoded)

decoded = Dense(vector_length, activation='sigmoid')(decoded)

autoencoder = Model(placeholder_input, decoded)

encoder = Model(placeholder_input, encoded)

########################### COMPILE AND FIT MODEL ###########################

autoencoder.compile(optimizer='adam', loss='binary_crossentropy')

autoencoder.fit(X_train,

X_train,

epochs=12,

batch_size=32,

shuffle=True,

verbose=0, validation_data=(X_test, X_test))

X = encoder.predict(X)

################### FIT KMEANS WITH AUTOENCODER VALUES ###################

for i in range(2, 10):

km = KMeans(n_clusters=i, random_state=0).fit(X)

clusters_km = km.labels_

print("Metrics KMEANS with %d clusters: \n" % (i))

print("Silhoutte: %f" % (silhouette_score(X, clusters_km, metric='euclidean')))

print("Davies: %f \n" % (davies_bouldin_score(X, clusters_km)))

######## FIT AGLOMERATIVE CLUSTERING WITH AUTOENCODER VALUES ##########

ag = AgglomerativeClustering(n_clusters=i).fit(X)

clusters_ag = ag.labels_

print("Metrics AGGLOMERATIVE with %d clusters: \n" % (i))

print("Silhoutte: %f" % (silhouette_score(X, clusters_ag, metric='euclidean')))

print("Davies: %f \n" % (davies_bouldin_score(X, clusters_ag)))
