# Supplementary material for: A Deep Learning Approach to Population Structure Inference in Inbred Lines of Maize
Source: Front Genet. 2020 Nov 24;11:543459. doi: 10.3389/fgene.2020.543459 (PMC7732446; doi:10.3389/fgene.2020.543459)
Supplement: Supplementary file 1 [file Data_Sheet_1.ZIP › Supplementary_Data_Sheet_S3.docx]

# Deep Autoencoders

Autoencoders are ML algorithms that are typically used as a pretraining step and dimensionality reduction. An autoencoder is an unsupervised artificial neural network with the main objective of learning a representation (encoding) for a dataset, by training the neural network to ignore the noise (Vincent et al., 2008). A standard autoencoder consists of two parts: encoder and decoder. In the encoder layer, the network learns to map the data to a lower-dimensional feature space. In the decoder layer, the network is responsible for reconstructing the coded, dimensionally reduced data.

The objective of the autoencoder is the generation of a reconstructed ***z*** of the input data ***x***, where ***z*** ≈ ***x*** by minimizing the loss function L(***x***, ***z***). This loss function can be defined as the mean squared error for continuous data (equation 1) or cross-entropy for discrete data (equation 2). In this study, it was minimized the cross-entropy loss between the input x and the reconstructed ***z***.

| $L\left( \boldsymbol{x},\mathbf{z} \right)=\left\vert\left\vert\boldsymbol{x}-\boldsymbol{z} \right\vert\right\vert^{2}$ | (1) |
| --- | --- |
| $L\left( \boldsymbol{x},\boldsymbol{z} \right)=-\sum_{k=1}^{d} \boldsymbol{x}_{k} log \boldsymbol{z}_{k}+\left( 1-\boldsymbol{x}_{k} \right) log\left( 1-\boldsymbol{z}_{k} \right)$ | (2) |

# K-Mean

K-mean is an unsupervised algorithm that works by clustering objects according to their features in K clusters (Macqueen 1967). The first step in K-mean is to choose a k value to set k centroids in the data space (random location). Subsequently, each object in the data space is assigned to its nearest centroid, and the positions of the centroids of each group are updated based on the minimization of the objective function. This function corresponds to the quadratic error, given by:

| $J=\sum_{j=1}^{k} \sum_{i=1}^{n} \left\vert\left\vert x_{i}^{\left( j \right)}-c_{j} \right\vert\right\vert^{2}$ | (3) |
| --- | --- |

where $\left| \left| \boldsymbol{x}_{\boldsymbol{i}}^{\left( \boldsymbol{j} \right)}\boldsymbol{-}\boldsymbol{c}_{\boldsymbol{j}} \right| \right|^{\boldsymbol{2}}$ is the distance measured from any point $\boldsymbol{x}_{\boldsymbol{i}}^{\left( \boldsymbol{j} \right)}$ to the nearest centroid $\boldsymbol{c}_{\boldsymbol{j}}$. The function J represents a measure of similarity of the n objects corresponding to each group. K-mean was implemented with Scikit-learn library.

# Hierarchical Clustering

HC method is an unsupervised algorithm that creates nested clusters by merging or dividing them successively. This hierarchy is represented as a tree or dendrogram. The root of the tree is the only group that gathers all the samples and the leaves are the groups with a single sample. Initially, in this algorithm, the objects belong to a list of unit sets S*_1_*, S*_2_*, ... S*_n_*, where a cost function is used to find the pair of sets S*_i_* and S*_j_*, from the closest list to merge. Once merged, they are removed from this setlist and replaced with S*_i_* ∪ S*_j_*. This process is carried out until all the objects are in a single group (Abbas 2008).

# Bayesian inference of population structure

The Bayesian clustering method implemented in the program InStruct (Gao et al., 2007) was used to infer the population structure by assuming an admixture model and correlated allele frequencies (Maldonado et al., 2019). Five independent runs were performed for each number of subpopulations (K), which was set from 1 to 9. For each K, 1x10^6^ Monte Carlo Markov Chain (MCMC) and a burn-in of 1x10^5^ iterations were performed. The optimal value of K was determined with the highest ΔK method proposed by Evanno et al. (2005), and the lowest value of Deviance Information Criterion (DIC) among the simulated groups (Gao et al., 2007).

# Evaluation metrics

The validation indexes are described as follows, which were computed using the Scikit-learn library in python language. Silhouette Coefficient (SC) analysis can be used to study the separation distance between the resulting clusters in order to evaluate the optimal number of clusters (Rousseeuw 1987). The SC has a range of [-1, 1], where a value near 1 indicates that the sample is far away from the neighboring clusters. A value of 0 indicates that the sample is on or very close to the decision boundary between two neighboring clusters and negative values indicate that those samples might have been assigned to the wrong cluster.

The Davies-Bouldin Index (DBI) evaluate the optimal number of clusters, in which a lower DBI (close to zero) indicates a better separation between clusters (Davies and Bouldin, 1979). This index can be defined as the average similarity between every cluster C*_i_* for *i*=1, …, *k* and its most similar one C*_j_*. In this way, the index is defined as the measure R*_ij_* that trades off: i) the average distance between every point of a cluster *i* and the centroid of that cluster (s*_i_*), ii) the distance between cluster centroids *i* and *j* (d*_ij_*).

A simple choice to construct a nonnegative and symmetric R*_ij_* is:

| $R_{ij}=\frac{s_{i}+s_{j}}{d_{ij}}$ | (4) |
| --- | --- |

Then, the DBI is defined as:

| $DBI=\frac{1}{k}\sum_{i=1}^{k} \max_{i\neq j} R_{ij}$ | (5) |
| --- | --- |
