# Supplementary figures and images for: A Deep Learning Approach to Population Structure Inference in Inbred Lines of Maize
Source: Front Genet. 2020 Nov 24;11:543459. doi: 10.3389/fgene.2020.543459 (PMC7732446; doi:10.3389/fgene.2020.543459)

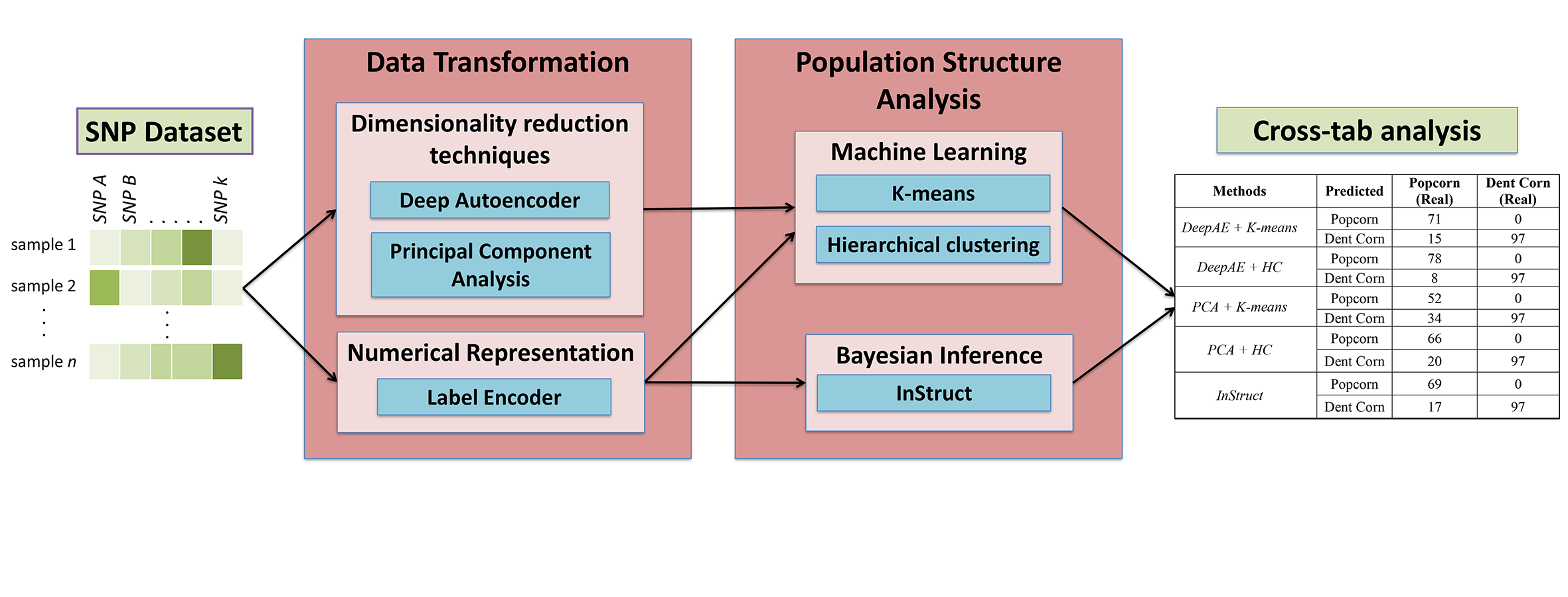

Supplement: Supplementary file 1 [file Data_Sheet_1.ZIP › Supplementary_Figure_S4.tif]
